# Screening procedures to identify problematic substance use in medical emergency department patients

**DOI:** 10.1186/1940-0640-7-S1-A79

**Published:** 2012-10-09

**Authors:** Michael Bogenschutz, Dennis Donovan, Cameron Crandall, Robert Lindblad, Raul Mandler, Harold Perl, Alyssa Forcehimes

**Affiliations:** 1 Department of Psychiatry, University of New Mexico, Albuquerque, NM, USA; 2 Alcohol & Drug Abuse Institute, University of Washington, Seattle, WA, USA; 3 Department of Emergency Medicine, University of New Mexico, Albuquerque, NM, USA; 4 EMMES Corporation, Rockville, MD, USA; 5 Center for Clinical Trials Network, National Institute on Drug Abuse, Bethesda, MD, USA; 6 Department of Prevention Research, National Institute on Drug Abuse, Bethesda, MD, USA; 7 Center on Alcoholism, Substance Abuse, and Addictions, University of New Mexico, Albuquerque, NM, USA

## 

Little is known about optimal screening processes for brief intervention (BI) targeting substances other than alcohol and nicotine. An ongoing multi-site trial conducted through the National Institute on Drug Abuse-Clinical Trials Network (NIDA-CTN) investigates the effectiveness of BI for people who use drugs (with or without alcohol use) presenting to medical emergency departments (EDs). Here we describe the screening procedures and present preliminary data on the screening process and characteristics of participants enrolled in the trial. Following triage, patients are selected through ED tracking logs, and demographic information, triage level, and presenting complaint are recorded. Patients are then invited to complete screening with an instrument including four tobacco screening questions, the three consumption questions of the Alcohol Use Disorders Identification Test (AUDIT-C), and the 10-item Drug Abuse Screening Test (DAST). A DAST score of three or more plus past 30-day use of the primary problem substance are required for enrollment. During the first six months of recruitment, a total of 5713 patients were selected for possible screening; 3731 completed screening, and 343 were randomized (6% of all selected). The sample is 64% male with a mean age of 39 (± 12), and is racially and ethnically diverse. Only 2% are college graduates, 9% are married, 10% have full-time jobs, and 79% have household incomes under $15,000. The most common primary drugs of abuse were cannabis (33%, used on an average of 18 out of the past 30 days), cocaine (26%, use on and average of 9 of the past 30 days), street opioids (26%, used on an average of 19 of the past 30 days), prescription opioids (6%, used on an average of 18 of the past 30 days), and methamphetamine (6%, used on an average of 7 of the past 30 days). Six percent of those selected for screening and 9% of those screened were enrolled in the study. Baseline data indicate that the screening procedures identified people with problems in multiple life domains who had heavy use of several types of drugs.

